# Long-Term Functional Recovery after Severe Traumatic Brain Injury with Type II Diffuse Injury

**DOI:** 10.1089/neur.2024.0052

**Published:** 2025-01-08

**Authors:** Marcia Harumy Yoshikawa, Sérgio Brasil, Davi Jorge Fontoura Solla, Robson Luís Amorim, Daniel Augustin Godoy, Angelos Kolias, Wellingson Silva Paiva

**Affiliations:** ^1^ Division of Neurosurgery, Department of Neurology, University of Sao Paulo, Brazil.; ^2^ Department of Intensive Care, Neurointensive Care Unit, Pasteur Hospital, Argentina.; ^3^ Division of Neurosurgery, Department of Clinical Neurosciences, University of Cambridge and Addenbrooke’s Hospital, UK.

**Keywords:** traumatic brain injury, outcome, caregiver burden, functional independence measure

## Abstract

This study aims to describe the late clinical outcomes of patients with severe traumatic brain injury (sTBI) and the risk factors associated with it. Patients were enrolled between April 2012 and January 2015 and followed until January 2022. The inclusion criteria were age 16–65 years, Glasgow Coma Scale ≤8 on admission, diagnosis of blunt TBI with Marshall diffuse injury type II on initial computerized tomography (CT), and alive at discharge. Clinical, laboratory, and radiological data from admission were collected. Glasgow Outcome Scale Extended (GOSE), Functional Independence Measure, and Zarit Burden Interview (ZBI) were assessed in the follow-up. Sixty-five patients were included, with a median follow-up time of 8 years. Nineteen (29.2%) patients had good recovery (GOSE 7–8), and 10 (15.3%) had moderate-to-severe sequelae (GOSE 4–6). Thirty-six (55.4%) patients died after discharge, and most of them in the first 3 months after discharge (*n* = 26; 72.2%). Despite the early mortality rate being the highest, the 6-month score is explained in the text (CRASH-CT) score on admission was not associated with death in the follow-up (*p* = 0.25). In the multivariate statistical analysis, only prothrombin time was associated with GOSE (*p =* 0.01). Twelve (41.3%) patients were independent for basic activities of daily living, and the most common cause of dependence was memory impairment (*n* = 12; 41.3%). The median ZBI score reported by caregivers was 23.5 (range 5–48), indicating mild overload. In this study, patients with sTBI sustaining Marshall II lesions had a significant mortality rate after discharge, and we found coagulation impairment as a potential predictor of poor outcomes. Around 30% experienced functional dependence and inability to return to social and work activities. Current instruments used to predict outcomes of TBI patients had poor predictive performance in this low- and middle-income country population, suggesting the need for new models to properly guide clinical decision-making and counseling family members.

## Introduction

Traumatic brain injury (TBI) is a medical and social condition of high importance worldwide, with an estimated incidence of 10 million new cases per year.^1,2^ In Brazil, the annual hospitalization rate due to TBI is 65.7 per 100,000 inhabitants, with young adults (20–29 years old) being the most affected age group and accounting for the highest number of in-hospital deaths.^3^ The economic impact of this condition is even more remarkable when considering the morbidity secondary to motor, cognitive, behavioral, and emotional sequelae in a population of working age.

The long-term outcomes of severe TBI (sTBI) have been extensively studied in high-income countries (HICs),^4–6^ and data from these studies have been used to create models to predict the clinical outcomes of TBI patients.^4–6^ The CRASH-CT score is one of the most validated prognostic models, which uses radiological and clinical data (including age, Glasgow Coma Scale [GCS], and pupil reactivity) to predict 14-day mortality and 6-month unfavorable outcomes of TBI patients.^7^

The Marshall CT is a descriptive classification of computerized tomography (CT) scan abnormalities that depict a relatively good accuracy of the risk of death within 12 months of a patient with TBI.^8–11^ The Marshall CT stratifies the severity of the lesion from I to VI: grades I–IV comprise diffuse injuries, and V–VI comprise mass lesions. Patients with diffuse injury II have midline shift ≤5 mm, absence of basal cistern compression, and high or mixed density lesion >25 mL.^12^ Most patients with Marshall type II diffuse injury are alive in the short term, with a survival rate in the first 3 months higher than 70%.^8–11^

Although data regarding long-term outcomes are crucial for medical decisions, resource allocation, and family communication, there are very few studies on the long-term effects of sTBI in low- and middle-income countries (LMICs). Most available studies are limited to mortality assessment within the first year of follow-up.^12–15^ Therefore, this study aimed to assess the clinical outcomes of sTBI patients with diffuse injury grade II on initial CT scans more than 7 years after hospital discharge. The primary outcome was the long-term Glasgow Outcome Scale Extended (GOSE), whereas the secondary outcomes included the Functional Independence Measure (FIM) and the caregiver burden measured by the Zarit Burden Interview (ZBI).

## Methods

### Ethical standards

This cohort study was performed in the Central Institute of Hospital das Clínicas da FMUSP (HC-FMUSP) and was approved by the Ethics and Research Committee of HC-FMUSP. All methods were performed following the relevant guidelines and regulations.

### Patient selection

This study enrolled patients from a tertiary university hospital admitted between March 2012 and January 2015. Subjects with (1) 16–65 years of age on admission, (2) GCS ≤8 on admission, (3) diagnosis of blunt TBI with Marshall diffuse injury II on initial CT scan, and (4) alive at discharge were included. Patients who experienced previous TBI episodes or had previous diagnoses of disabling neurological diseases were excluded.

### Data collection

Clinical, laboratory, and radiological data from admission were obtained from a digital database created to collect systematized information from patients admitted to the intensive care unit (ICU) between March 2012 and January 2015. Clinical and laboratory information present in the database was obtained at the time of admission to the ICU. Radiological findings were collected using the Philips’ iSite PACS system (Philips Electronics, USA, 2006). Long-term outcomes were assessed between October 2021 and January 2022 using standardized questionnaires. The outcomes assessed included GOSE (primary outcome) and FIM (secondary outcome). During follow-up interviews, caregivers were asked to join the study by answering the 22-item ZBI.

### Database

The database included TBI patients admitted to a trauma ICU from March 2012 to January 2015. Patients were admitted to the emergency unit and initially evaluated by the surgical team, following the recommendations of advanced trauma life support. The neurosurgery team evaluated patients with a clinical–radiological diagnosis of TBI and followed the Brain Trauma Foundation Guidelines. Patients admitted to the ICU, whether undergoing surgery or not, were recruited and had their data recorded. The ICU admission criterion was any patient with TBI and intracranial tomographic abnormality. Access to the database was restricted to individuals directly linked to research, maintaining the protection and confidentiality of participants’ data.

### Statistical analysis

Categorical data were presented as absolute numbers (with percentages), parametric data as mean and standard deviation, and nonparametric data as medians and interquartile range. Regarding statistical analysis, the unpaired Student’s *t* test or Mann–Whitney test for continuous variables was used to assess the association of variables with outcomes, and the chi-square or Fisher’s exact test for categorical variables. We used the Shapiro–Wilk test to assess the normality of the data. Variables significantly associated with the primary outcome in univariate analysis (*p* < 0.05) were included in the statistical model (multivariate analysis). Given the limited number of patients in each stratum of GOSE, the primary outcome was dichotomized into poor outcome (GOSE 1–3) and favorable outcome (GOSE 4–8). Data were analyzed using the SPSS 29.0 program.

## Results

**FIG. 1.**
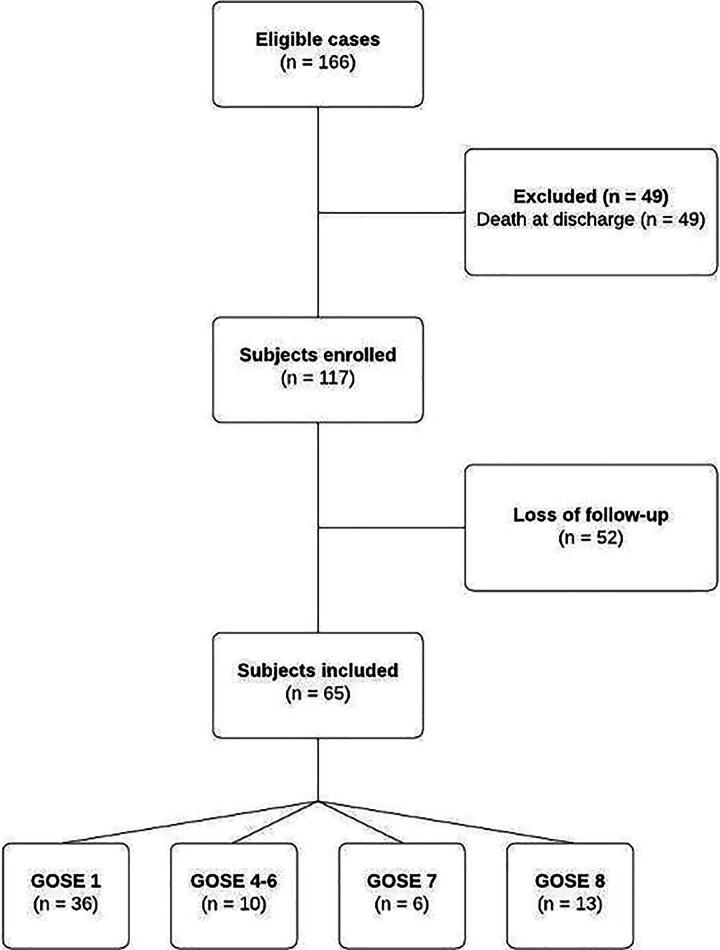
Flowchart of study population selection.

A total of 117 patients were enrolled, and 52 (44.5%) lost to follow-up after hospital discharge. Sixty-five patients were included (Fig. 1) in the final analysis, with a mean age of 42.51 ± 18.25 years on admission. Most patients were male (*n* = 56; 86.1%), and 13 (20%) received neurosurgical treatment. All participants were admitted to the ICU, with a mean stay of 22.32 ± 20.22 days and a median Simplified Acute Physiology Score 3 of 53 ± 19.5. The mean length of hospitalization was 38 ± 3.82 days (*p* = 0.09). Tables 1 and 2 present detailed clinical, laboratory, and radiological information from the ICU admission of the study population. Regarding the subjects who were lost to follow-up, most were male (*n* = 49; 94.2%) with a mean age of 31.63 ± 12.34 years, a median GCS on admission of 6 ± 4, and a mean prothrombin time of 1.33 ± 0.31.

**Table 1.** Baseline Characteristics on Intensive Care Unit Admission and Their Association with Glasgow Outcome Scale Extended Score (Univariate Analysis)

Variable	Total (*n* = 65)	*p* value
**Age^c^**	42.51 ± 18.25	0.02
**Male**	56 (86.1%)	0.72
**SAPS3**	54.27 ± 14.79	0.41
**Reactive pupils^a^**	—	0.62
None	1 (1.5%)	—
One	7 (10.7%)	—
Both	52 (80%)	—
**Hypoxia**	40 (61.5%)	0.57
**Hypotension**	18 (27.7%)	0.6
**Extracranial trauma**	58 (89.2%)	0.12
**Orotracheal intubation**	38 (58.4%)	0.59
**Glasgow Coma Scale^d^**	3 ± 4	0.009
**Glasgow Motor Score^b^, ^d^**	5 ± 2	<0.0001
1	10 (15.3%)	—
2	4 (6.1%)	—
3	4 (6.1%)	—
4	8 (12.3%)	—
5	27 (41.5%)	—
6	10 (15.3%)	—

^a^ Five (7.6%) patients did not have information about pupil reactivity on admission.

^b^ Two (3%) patients did not have information about motor score on admission.

^c^ Values presented as mean ± standard deviation.

^d^ Values presented as median ± interquartile range.

SAPS3, Simplified Acute Physiology Score-3.

**Table 2.** Laboratory Data on Intensive Care Unit Admission and Its Association with Glasgow Outcome Scale Extended Score in the Follow-Up (Univariate Analysis)

Variable	Mean ± SD	*p* value
**Prothrombin time**	1.3 ± 0.24	0.02
**Thromboplastin time partial test**	1.15 ± 0.31	0.83
**Hemoglobin**	11.2 ± 2.23	0.39
**Hematocrit**	33.4 ± 6.44	0.37
**Platelets**	184.36 ± 67.14	0.68
**Creatinine**	1.23 ± 1.21	0.16
**C-reactive protein**	78.46 ± 91.99	0.51

**FIG. 2.**
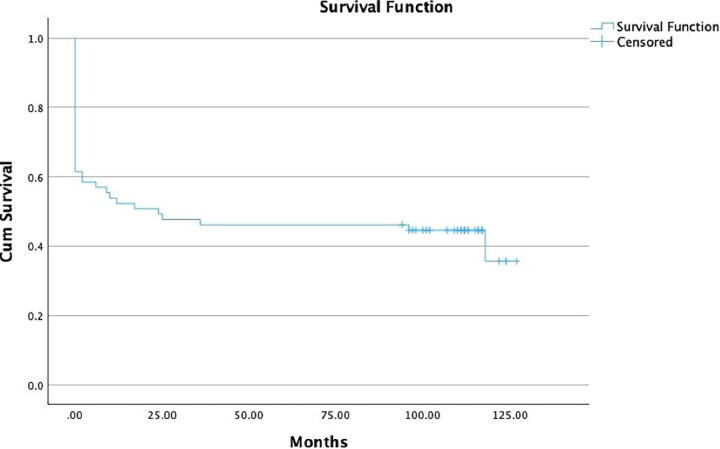
Survival function of the study population.

The median follow-up time was 8 years. Thirty-six (55.4%) patients died during follow-up. The mean age on admission of patients who died during the follow-up was 47.08 ± 18.36 years. Most of the deceased patients (*n* = 26; 72.2%) passed away within the first 3 months after discharge, 3 (8.3%) patients died between 3 and 12 months after discharge, 5 (13.8%) died between 12 and 24 months after discharge, and 2 (5.5%) died after 24 months of discharge (Fig. 2). Among those who survived (*n* = 29), the mean age on admission was 36.89 ± 16.39 years. Thirteen patients (44.8%) had complete recovery (GOSE 8), 6 (20.6%) had good recovery (GOSE 7), and 10 (34.4%) had moderate-to-severe sequelae (GOSE 4–6; Table 3).

**Table 3.** Functional Outcome and Caregiver Burden in the Follow-Up

Variable	Total
**GOSE**	**65 (100%)**
Death (GOSE 1)	36 (55.4%)
Moderate-to-severe sequelae (GOSE 3–6)	10 (34.4%)
Good recovery (GOSE 7)	6 (9.2%)
Complete recovery (GOSE 8)	13 (20%)
**FIM**	**29 (100%)**
Dependent (18–108)	4 (6.1%)
Independent (109–126)	25 (38.4%)
**ZBI score**	**8 (100%)**
No overload (<20)	3 (37.5%)
Mild overload (21–40)	4 (50%)
Moderate overload (41–60)	1 (12.5%)

FIM, Functional Independence Measure; GOSE, Glasgow Outcome Scale Extended; ZBI, Zarit Burden Interview.

Only 12 (41.3%) patients were entirely independent for basic activities of daily living (BADL) according to FIM (Table 3). The median FIM score was 124 ± 6. The most common cause of dependence was memory impairment (*n* = 12; 41.3%), followed by inability to dress lower (*n* = 9; 31%) and upper (*n* = 8; 27.5%) body with complete independence, climbing stairs (*n* = 8; 27.5%), and problem-solving impair (*n* = 8; 27.5%).

Of 29 patients, 9 had caregivers, and 8 agreed to participate in the research. All caregivers were patients’ relatives. The median FIM score of patients with caregivers was 114 (range 21–125). The median ZBI score (ZBIs) was 23.5 (range 5–48). Three (37.5%) caregivers reported no overload (ZBIs <22), four (50%) reported mild overload (ZBIs between 22 and 44), and only one (12.5%) reported moderate overload (ZBIs between 44 and 66; Table 3).

**FIG. 3.**
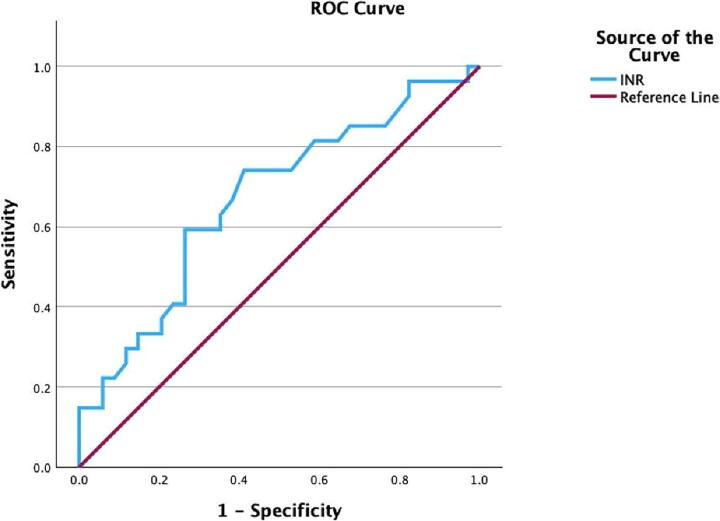
Receiver operating characteristic (ROC) curve of prothrombin time (INR) for prediction of long-term survival. Area under the curve AUC 0.670; confidence interval 95% 0.532–0.808.

The univariate statistical analysis demonstrated that the GCS on admission, Glasgow Motor Score, and prothrombin time International Normalized Ratio (INR) were associated with the GOSE score in the follow-up (*p =* 0.009, *p* < 0.0001, and *p =* 0.02, respectively). The CRASH-CT score was not associated with death after discharge (*p* = 0.25). In the multivariate analysis, only INR maintained a significant association with survival in the follow-up (*p* = 0.01). However, INR demonstrated a poor predictive performance (AUC 0.670; confidence interval [CI] 95% 0.532–0.808; Fig. 3).

## Discussion

This is the first cohort study about sTBI in a Brazilian adult population to assess functional outcomes ≥7 years post-trauma. In this study, most patients died during follow-up. Among those who survived, more than one-third were unable to return to work or social activities, and more than half needed assistance for BADL. Coagulation impairment had a significant association with outcomes in the follow-up.

De Silva et al.^16^ assessed the 6-month outcome of TBI patients from 46 countries, concluding that patients with sTBI from LMIC are more likely to die in the first 6 months after injury than patients from HIC, which might be associated with differences in rescue time and medical care. In tandem with that, we found a high mortality rate (55.4%) in the follow-up, especially in the first 3 months after discharge, which was not individually associated with the CRASH-CT score or its clinical and radiological variables.

However, it is important to highlight that our cohort demonstrated a significant rate of loss to follow-up, and those who were lost tended to be younger and have better neurological conditions at the time of admission. Long-term follow-up after a TBI is known to be challenging.^17,18^ Smith et al.^18^ investigated this topic using data from 13 LMICs and identified two potential reasons for loss to follow-up in these settings: socioeconomic barriers to continued access to health care and the perception of satisfactory recovery by patients and their families after motor recovery. Given these barriers, individuals who are economically disadvantaged and who have partial recovery are more likely to be underrepresented in long-term TBI studies in LMIC. Additionally, the psychological and behavioral effects of TBI are further reasons for loss to follow-up due to memory impairment, resulting in forgetting appointments, lack of willingness to cooperate, and lack of concern about their health condition.^18^ Therefore, the analysis of the included population in this study may have overestimated the true mortality rate of sTBI patients while underestimating less severe, yet burdensome sequelae.

Only INR was associated with GOSE in the multivariate analysis of the clinical and laboratory data from ICU admission. Previous studies have suggested the role of coagulability in the mortality and disability of TBI patients, mainly due to the higher risk of intracranial bleeding.^19–21^ Coagulability disturbances are common after TBI, which may occur from minutes to hours after injury, and in 30% of the cases, it can extend for more than 72 hours.^22^ Solla et al.^23^ have demonstrated that the inclusion of coagulopathy markers increases the prognostic accuracy of the CRASH-CT score, with altered results of platelet count, INR, or Activated Partial Thromboplastin Time (aPTT) ratio associated with doubled odds of death in 14 days post-trauma.

De Silva et al.^16^ also reported a decreased risk of disability—measured through the Glasgow Outcome Scale—in patients from LMIC who suffered mild or moderate TBI, pointing out cultural particularities and differences in the definition of disability as potential reasons for this finding. Indeed, we found a mismatch between the functional recovery measured by GOSE and the independence for BADL measured by FIM, which suggests a different perception of disability in the study population with mild sequelae.

According to the FIM criteria, few subjects were dependent on activities of daily living (FIM 18–108); however, several experienced critical cognitive and physical limitations years post-trauma, which were reflected in the inability to return to previous social and work activities reported by the GOSE scores. In our cohort, memory disability was the most frequent source of dependence. In fact, TBI is known to impair memory, primarily its working domain, which is the ability to temporarily hold, process, and manipulate information and an essential ability in planning and performing tasks.^24^ Experimental models demonstrated that TBI affects working memory by limiting the ability to encode new information in the acute post-injury period and to keep information chronically.^25–28^

The motor, emotional, and cognitive limitations secondary to TBI result in caregiver burden.^29,30^ The 22-item ZBI assessed the burden on quality of life, psychological health, social and family relationships, financial issues, and feelings of shame and guilt. The score ranges from 0 to 88: scores 0–20 indicate little-to-no burden, 21–40 mild-to-moderate burden, 41–60 moderate-to-severe burden, and 61–88 severe burden.^31^ In our study, although many patients reported significant dependence on specific activities, few patients had a caregiver, and among the caregivers, most of them reported mild or no burden. This finding contrasts with previous studies about TBI caregivers in Latin America, which demonstrated that they experience marked reductions in mental health, mainly due to poor social and emotional self-regulation of TBI patients at discharge.^16,32–34^ A potential reason for this finding is the high mortality rate in the follow-up, which likely included patients who were more dependent and who accounted for a more significant burden.

## Limitations

The main limitation of this study is the small sample size due to the loss to follow-up. The restricted number of patients lowered the study’s statistical power, which was partially addressed by dichotomizing the primary outcome. Performing long-term follow-ups in LMICs is particularly challenging due to the difficulty of contacting patients, which reflects a lack of standardized rehabilitation programs for TBI patients. Additionally, the study population was not drawn from a larger TBI population but from a single tertiary care hospital, limiting the study’s external validity.

## Conclusions

Patients with sTBI sustaining Marshall II lesions have a significant mortality rate after discharge, and we found coagulation impairment as a potential predictor of poor outcomes. Additionally, around one-third of sTBI patients have significant functional dependence and are unable to return to previous social and work activities years following trauma. Despite these poor results, participants demonstrated surprisingly low rates of caregiver burden. Finally, current instruments used to predict outcomes of TBI patients had a poor predictive performance in this LMIC population, suggesting the need for new models to properly guide clinical decision-making, resource allocation, and counseling family members.

## Data Availability

The datasets used and/or analyzed during the current study are available from the corresponding author upon reasonable request.

## Abbreviations Used

AUC: Area Under the Curve
BADL: Basic Activities of Daily Living
CT: Computed Tomography
FIM: Functional Independence Measure
GCS: Glasgow Coma Scale
GOSE: Glasgow Outcome Scale Extended
HICs: High-Income Countries
ICU: Intensive Care Unit
INR: International Normalised Ratio
LMICs: Low- and Middle-Income Countries
ROC: Receiver Operating Characteristic
sTBI: Severe Traumatic Brain Injury
TBI: Traumatic Brain Injury
ZBI: Severe Traumatic Brain Injury
ZBIs: ZBI Score
